# Geographical Discrimination of Croatian Wines by Stable Isotope Ratios and Multielemental Composition Analysis

**DOI:** 10.3389/fnut.2021.625613

**Published:** 2021-03-04

**Authors:** Renata Leder, Ivana Vladimira Petric, Josipa Jusup, Mara Banović

**Affiliations:** ^1^Department of Physico-Chemical Testing, Center for Viticulture, Enology and Edible Oils Analysis, Croatian Agency for Agriculture and Food, Zagreb, Croatia; ^2^Department for Authentic Products, Center for Viticulture, Enology and Edible Oils Analysis, Croatian Agency for Agriculture and Food, Zagreb, Croatia; ^3^Primevigilance d.o.o., Zagreb, Croatia; ^4^Department of Food Engineering, Faculty of Food Technology and Biotechnology, University of Zagreb, Zagreb, Croatia

**Keywords:** Croatian wines fingerprint, elements, geographical origin, inductively coupled plasma optical emission spectroscopy, isotope ratio mass spectrometry, stable isotopes

## Abstract

The δ^18^O and δ^13^C (analyzed by isotope ratio mass spectrometry, IRMS) and concentration of 22 selected elements (analyzed by inductively coupled plasma—optical emission spectrometry, ICP-OES) in 190 Croatian microvinified and commercial wine samples from continental and coastal winegrowing areas and from three viticultural zones (B, CI, and CII) were measured to investigate whether multivariate statistical methods could provide the fingerprint for geographical origin determination. The highest power for discrimination of wines produced in Croatian winegrowing areas was achieved by general discriminant analysis (GDA) showing correct classification of 97.9% of all investigated samples, 100.0% of microvinified samples and 84.8% of commercial samples in the cross-validation matrix. The most significant markers for discrimination of coastal and continental areas found by GDA were δ^18^O and Co, followed by K, Rb, Sn, Li, and δ^13^C in descending order. GDA showed higher levels of correctly classified samples from three viticultural zones in Croatia if only microvinified samples were employed in the analysis (94.9%) than for all samples together (86.3%) or for commercial samples (66.1%) in the cross-validation matrix. The discrimination of viticultural zones B, CI, and CII in Croatia was achieved by δ^18^O, Co, Rb, Li, K, and Sn. The results obtained showed that the relationships between the isotopic ratios and concentrations of different considered elements combined with appropriate statistical model represent a powerful tool in discrimination of wines produced in different Croatian winegrowing areas.

## Introduction

The adulteration of food and beverages is a growing global problem. Consumer awareness of the food safety importance has been steadily increasing in recent years as well as activities that include adulteration of food products for economic gain (1, 2). Following these trends, analytical methods for determination of the authenticity of food products, including wine, are also constantly being developed and upgraded accordingly (3, 4). Appropriate chemometric analysis of the data provided by those methods are needed and proposed for wine (5–7) and other food types, i.e., honey (8), cheese (9), and meat (10). Authenticity and commercial value of wine are often associated with geographical origin, and some countries or regions are known for producing high commercial value wines (3). Wine is a product that is often adulterated by the addition of sugar and/or water, as well as through intentional mislabeling of origin for economic gain (6, 11, 12). Hence, the use of analytical methods to verify the declared composition and origin is of high-interest both for wine producers and consumers (13, 14). This is also increasingly recognized in Croatia (15–19), where viticulture and winemaking represent a significant economic activity, especially through the growing tourism industry (20).

The relationship between the isotope data of wine and physical variables related to the climate and geography of the production area is a very interesting topic, as is evident in many published papers in the last 20 years (5, 11, 21–28).

Just like the stable isotopes, mineral elements are also considered to be good indicators of geographical origin of wine since they are neither metabolized nor modified during the wine production (29). Distinction of wine region through trace element composition is due to their close connection with their transfer from rock to soil and from soil to grape. The multi-element composition of wine is strongly influenced by the solubility of inorganic compounds in the soil and, in principle, the multielement composition of wine reflects the soil geochemistry of the grape growing region (30). Recent research has been conducted to determine the geographic origin based on the composition of the elements assuming that the chemical composition of the wine reflects the soil composition (31), in which case their determination enables the establishment of a “fingerprint” for each element and creates the possibility of establishing a link between wine and their geographical origin (26). The potential of multielement “fingerprint” techniques to identify the geographical origin of wine was established in many investigations in different countries: Portugal (32, 33), Italy (34, 35), Slovakia (36), Croatia (37, 38), Spain (39–41), Romania (42), Cyprus (7, 14), Slovenia (4), Serbia (43), Macedonia (44), Ukraine (45), Turkey (46), Argentina (47), South Africa (31, 48, 49), and California (50), USA.

Numerous researchers applied combined isotopic and multielement methods to determine the geographical origin of wine. One of the oldest such studies was carried out on French wines from the Bordeaux region (51). The characterization of Swiss vineyards using isotope data in combination with trace elements and classical parameters has also demonstrated the possibilities of multidimensional statistical data processing (52). IRMS, ICP-OES, and NMR analysis of authentic wines that are part of a Cypriot bank of authentic wines as well as analysis of Cypriot commercial wines have been carried out and the observed variations in isotopes and elements were compared with grape varieties, environmental conditions, and geographical origin (7, 14). The possibilities of isotope and multielement techniques as “fingerprints” have been explored in regional differentiation of Romanian wines for 2 years of harvest and various autochthonous and introduced varieties (53). The differentiation of wine samples from the border areas of Austria, Czech Republic, Slovakia (and from Serbia) was investigated by applying different techniques (e.g., IRMS, NMR, ICP-MS, ICP-OES, EPR, HPLC, UV-VIS, etc.) showing promising possibilities for provenance studies (54).

Research to determine the geographical origin of wine has not only been conducted in Europe. Articles published by Argentine (55), Brazilian (56), Chinese (57), and American (58) authors are also available in the scientific literature. The most important conclusion of these studies is that the combined application of isotopic and multi-element methods with multivariate statistical methods will provide a promising statistical model for the classification of wines with regards to their geographical origin.

There are few published studies on determining the geographical origin of Croatian wines with regard to isotopic data (24), elemental profiles (37, 38, 59), or some other aspects of wine quality, i.e., polyphenolic composition (17). Present work is the first study to combine isotopic and multielemental methods for discrimination of Croatian wines according to their geographical origin.

**Figure 1:**
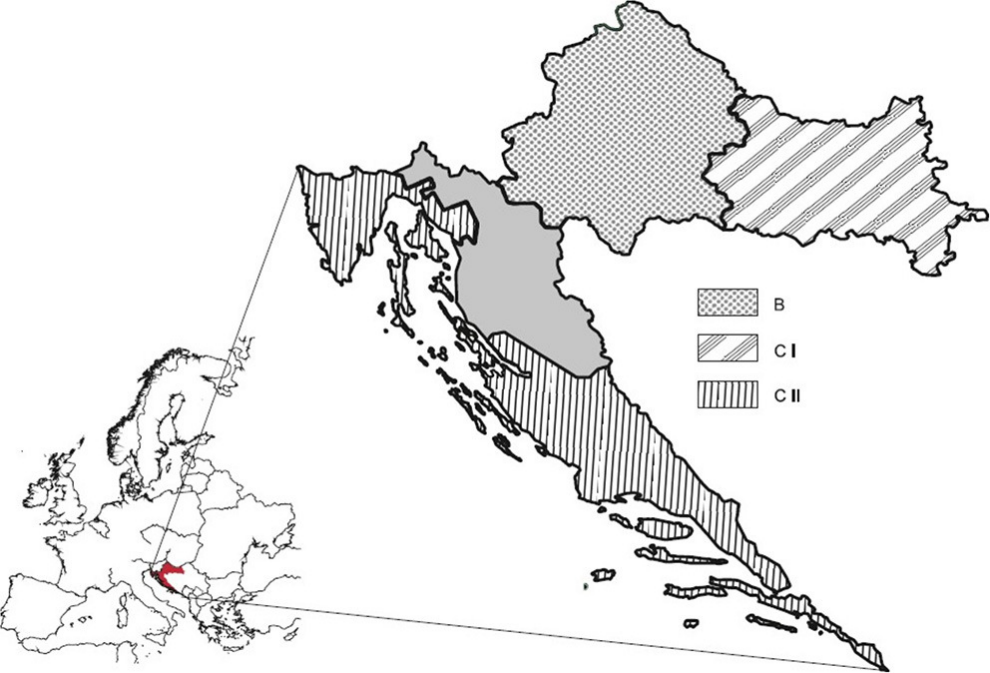
Croatian winegrowing zones according to EU Regulation (63). Continental Croatia = zone B (Croatian Uplands) + zone CI (Slavonia and Croatian Danube). Coastal Croatia = zone CII = Croatian Istria and Kvarner + Dalmatia. Gray area, no winegrowing region.

The geographic position of Croatia is a meeting point of a continental climate in the eastern and central parts of the country, and the Mediterranean climate in the southern, coastal areas. It is divided into three viticulture climate zones (B, CI, and CII; Figure 1) according to the Winkler (60) division system and into four winegrowing regions or geographical indications (61), which include 16 protected denominations of origin (PDO) registered at database for EU geographical indications eAmbrosia (62).

Temperature based bioclimatic Winkler index (WI) uses a growing degree base of 10°C (growing degree-days, GDD) and correlate viticulture with the climate through five viticulture regions (60). Accordingly, zone B (1391-1670 GDD or WI Region II) corresponds to the wine region of Croatian Uplands. The zone CI (1671-1940 GDD or WI Region III) appertains to the wine region of Slavonia and Croatian Danube. Zone CII (1941-2220 GDD or WI Region IV) includes two wine regions: Croatian Istria and Kvarner, and Dalmatia.

Croatia joined the EU in 2013 and consequently participates in the EU Wine Isotopic Databank in order to comply with EU legislation (64, 65). Following these requirements, we produced and analyzed microvinified wines that are a part of the Croatian national and EU Wine Databank bank as well as commercial wines from Croatian wine producing regions. δ^18^O of wine water and δ^13^C of wine ethanol were determined by Isotope Ratio Mass Spectrometry (IRMS) and concentrations of Al, As, B, Ba, Ca, Cd, Co, Cr, Cu, Fe, K, Li, Mg, Mn, Mo, Na, Pb, Rb, Sn, Sr, V, and Zn by Inductively Coupled Plasma—Optical Emission Spectrometry (ICP-OES). The aim of this study was to evaluate the obtained isotopic and multielement data by appropriate statistical methods in order to identify suitable geographical origin markers of Croatian wines and to obtain a chemometric tool for discrimination of wines produced in different Croatian winegrowing areas and zones.

## Materials and Methods

### Wine Samples

One-hundred and ninety wines of two vintages (2015 and 2016) were analyzed. In total, 78 samples were a part of the Croatian bank of authentic wines produced by microvinification in accordance to the EU legislation (65), and 112 wines were conventionally produced and obtained by the Croatian Agency for Agriculture and Food, Center for Viticulture, Enology, and Edible Oils Analysis after the procedure of placing the wine on the Croatian market. Both indigenous and international white and red vine varieties were represented among the samples. The majority of these wines were monovarietal and evaluated as top quality after physicochemical analysis and sensory evaluation. The following parameters were determined: relative density, alcoholic strength, total dry extract, reducing sugars, pH, total acidity, volatile acidity, ash, free, and total sulfur dioxide. Obtained results were in accordance to the specifications of declared PDO registered at database for EU geographical indications eAmbrosia (62). The limits of tested parameters for Croatian top quality PDO wines listed in specifications are: alcoholic strength ≥ 8.5% (v/v) for zone B wines, and ≥9.0 % (v/v) for the wines from CI and CII zones; reducing sugar-free extract ≥ 18, 19, and 20 g L^−1^ (for white, rose, and red wine, respectively); total acidity (as tartaric acid) > 3.5 g L^−1^; volatile acidity (as acetic acid) ≤ 1.1 g L^−1^ for white and ≤ 1.2 g L^−1^ for red wines; ash content ≥ 1.5, 1.6, and 1.8 g L^−1^ (for white, rose, and red wines, respectively). With regard to the aspect of food safety, it is important to emphasize that the concentration of SO_2_ in all samples did not exceed the limits: 150 mg L^−1^ for red and 200 mg L^−1^ for rose and white wines with the residual sugar content ≤ 5 g L^−1^; and 200 mg L^−1^ for red and 250 mg L^−1^ for rose and white wines with the residual sugar content > 5 g L^−1^. Details about the origin of wines according to the winegrowing areas, zones, regions, type of production, harvest, and varieties are given in Table 1.

Table 1Geographical areas, wine-growing zones, and regions, type of production (microvinified—A, commercial—C), harvest (2015 and 2016), and varieties (indigenous varieties are marked with an asterisk) of the investigated wine samples (Σn = total number of samples).

**Table:** 

**Area**	**Continental Croatia =** **(zone B + zone CI)**	**Coastal Croatia** **=** **zone CII**	**Σ*n***
*n* (Area)	120	70	**190**
*n* (A)	42	36	**78**
*n* (C)	78	34	**112**
*n* (2015)	53	38	**91**
*n* (2016)	67	32	**99**

**Table:** 

**Zone**	**B**	**CI**	**CII**		
**Region**	**Croatian Uplands**	**Slavonia and Croatian Danube**	**Croatian Istria and Kvarner**	**Dalmatia**	
*n* (Region)	78	42	29	41	**190**
*n* (A)	25	17	13	23	**78**
*n* (C)	53	25	16	18	**112**
*n* (2015)	31	22	16	22	**91**
*n* (2016)	47	20	13	19	**99**
White wine varieties (*n*)	Chardonnay (9), Gewürztraminer (3), Grüner Sylvaner (5), Kraljevina^*^ (1), Moscato Giallo (1), Müller Thurgau (1), Pinot Blanc (4), Pinot Gris (5), Riesling Italico (47), Rhein Riesling (7), Sauvignon Blanc (8), Škrlet^*^ (2), mixture of white varieties (1).		Bogdanuša^*^ (1), Cetinka^*^ (1), Chardonnay (6), Malvazija istarska^*^ (8), Maraština^*^ (1), Moscato Giallo (1), Pošip bijeli^*^ (6), Vugava^*^ (1), Žlahtina^*^ (1).		**120**
Red wine varieties (*n*)	Blaufränkisch (5), Cabernet Sauvignon (8), Merlot (9), Pinot Noir (1), Zweigelt (2), mixture of red varieties (1).		Babić^*^ (1), Cabernet Franc (1), Cabernet Sauvignon (2), Merlot (14) Plavac mali crni^*^ (23), Plavina^*^ (1), mixture of red varieties (2).		**70**

### Sample Preparation for δ^13^C Measurement

Determination of alcoholic strength of the wine samples was performed by electronic density meter coupled with near infrared spectrometer (DMA 4500 and Alcolyzer, Patent Anton Paar® (66); Anton Paar, Austria). Wine samples with a volume of 200 mL were distillated at ADCS—Automated Distillation Control System (Eurofins, Nantes, France), operated by the ADSC V1.1.9.0 software. Karl Fischer DL31 volumetric titrator (Mettler Toledo, Greifensee, Swizterland) operated by LabX light titration v2.6 software (Mettler Toledo, Greifensee, Swizterland) was used for the determination of the distillate water content (% w/w) in all obtained distillates in order to calculate the alcoholic strength (% w/w) and yield of each performed distillation. Eurokarl Windows v.1.0.0.0 software (Eurofins, Nantes, France) was used for transfer of the obtained alcoholic strength data to the ADSC V1.1.9.0 software (Eurofins, Nantes, France). Reagents used for the Karl-Fischer titration were Titrant 5, Solvent and 1% water standard for the standardization procedure of the solvent were obtained from Merck (Darmstadt, Germany). The requirements for the distillation procedure are described in the OIV method (OIV-MA-AS311-05:R2011) (67).

### Sample Preparation for ICP-OES Measurements

Residue of wine after obtaining the ethanol for δ^13^C measurement by ADCS distillation was used as described by Miloš et al. (68). The residue was returned to its initial volume and diluted 1:1 by 2% (v/v) HNO_3_.

### Sample Preparation for δ^18^O Measurement

No sample preparation was required.

### ICP-OES Measurements

The determination of 22 elements was conducted by 2000 Dual View Optima ICP-OES (Perkin Elmer, Shelton, Connecticut, USA) equipped with a Meinhard spray chamber (Meinhard, Golden, Colorado, USA), nebulizer, and peristaltic sample delivery system. The instrument was controlled by the ICP WinLab 1.35 Perkin Elmer software. The flow conditions for plasma gas, auxiliary gas, and nebulizer gas were 15.0 L min^−1^, 0.2 L min^−1^, and 0.8 L min^−1^, respectively. Radio frequency generator power was set at 1,300 W. Samples were analyzed by calibration curve method including the internal standard. Operating conditions of the used method were previously published (69).

The 60 % (v/v) ultrapure HNO_3_ (Merck, Darmstadt, Germany) was used diluted to 2% (v/v) by ultrapure water (18 MΩ cm^−1^ resistivity, Simplicity, Millipore, Molsheim, France) and used as blank, to prepare appropriate stock and calibration solutions and to dilute the samples. A 1 g L^−1^ ICP grade standard solution of yttrium (Perkin Elmer, Waltham, Massachusetts, USA) was used as an internal standard at the concentration of 100 μg L^−1^. Multi-element standards were prepared in-house by mixing of certified, traceable, ICP grade single element standards: 1 g L^−1^ of B and Cr (Acros Organics, New Jersey, USA), As, Ca, Cd, Mg, Mo, Na, Pb, Zn, and 10 g/L of K (Perkin Elmer, Waltham, Massachusetts, SAD), 1 g L^−1^ of Al, Ba, Co, Cu, Fe, Li, Mn, Rb, Sn, Sr, and V (Reagecon, Shannon, County Clare, Ireland). To eliminate potential contamination, all glassware, and polypropylene storage bottles were rinsed by HNO_3_ (2% v/v), and three times by ultrapure water, and allowed to dry before use.

Calibration was performed for each element at appropriate level (Table 2) and limits of detection and quantification were calculated as well as the recovery and measurement uncertainty (70, 71). The control charts of the standard reference material were used through the study period to ensure the quality of measurement results.

**Table 2:** Ranges of calibration, limits of detection (LOD), and limits of quantification (LOQ) expressed as concentration in matrix, recovery (%), and expanded measurement uncertainty (%) for all elements.

**Element (γ)**	**Calibration** **range (γ)**	**LOD (γ)**	**LOQ (γ)**	**Recovery** **(%)**	**Measurement uncertainty** **U (%)**
Al (mg L^−1^)	0.1–2.0	0.0004	0.0015	93	5
As (μg L^−1^)	15–300	9	28	97	11
B (mg L^−1^)	0.25–5.0	0.001	0.002	94	12
Ba (mg L^−1^)	0.05–1.00	0.00001	0.00005	99	5
Ca (mg L^−1^)	5.0–100.0	0.004	0.014	101	15
Cd (μg L^−1^)	1–20	0.3	0.9	101	4
Co (μg L^−1^)	0.5–10.0	0.4	1.2	106	11
Cr (μg L^−1^)	0.5–10.0	0.3	1.2	106	16
Cu (mg L^−1^)	0.05–1.00	0.0004	0.0013	98	5
Fe (mg L^−1^)	0.5–10.0	0.003	0.009	101	7
K (mg L^−1^)	100–2,000	0.05	0.16	106	8
Li (μg L^−1^)	1–20	0.004	0.014	93	7
Mg (mg L^−1^)	5–100	0.002	0.005	99	7
Mn (mg L^−1^)	0.25–5.00	0.00004	0.00014	101	6
Mo (μg L^−1^)	0.5–10	0.1	0.2	107	33
Na (mg L^−1^)	1.0–20.0	0.00	0.01	98	7
Pb (μg L^−1^)	15–300	5	16	101	5
Rb (mg L^−1^)	0.25–5.00	0.0003	0.0009	96	9
Sn (μg L^−1^)	0.05–1.00	0.004	0.012	99	5
Sr (mg L^−1^)	0.05–1.00	0.000004	0.000014	100	7
V (μg L^−1^)	5–100	0.3	1.1	107	8
Zn (mg L^−1^)	0.25–5.00	0.0005	0.0018	101	7

### IRMS Measurements

IRMS measurements were performed by IRMS Delta V Plus (Thermo Fischer Scientific, Bremen, Germany) coupled to Gasbench and Elemental Analyzer FlashEA 1112 Series, for δ^18^O and δ^13^C measurements, respectively. Instruments were controlled by the Isodat 3.0 software (Thermo Fischer Scientific, Bremen, Germany). The isotopic ratios of ^13^C/^12^C and ^18^O/^16^O are expressed in the delta notation, δ^13^C and δ^18^O, respectively, as part per thousand (‰). Determination of stable isotope ratio of δ^18^O in wine water was performed as described in the OIV method (OIV-MA-AS2-12:R2009) (67) after equilibration with helium and CO_2_ mixture at 24 ± 1°C for 24 h. The samples were analyzed against in house reference material calibrated by the certified reference materials VSLAP2 and VSMOW2 obtained at International Atomic Agency, Vienna, Austria. Determination of stable isotope ratio of δ^13^C in obtained ethanol was performed as described in the OIV method (OIV-MA-AS312-06:R2001) (67). The samples were measured against the certified reference material BCR-656 (Institute for Reference Materials and Measurements, Geel, Belgium). Chemicals used for filling the combustion reactor for conversion the sample ethanol in carbon dioxide were copper (II) oxide, silver cobaltous/cobaltic oxide, and chromic (III) oxide (Thermo Fischer Scientific, Bremen, Germany). The quality of measurement results was validated by control charts of appropriate reference materials, which were recorded during the study and confirmed by participating to the interlaboratory comparisons organized by Eurofins (Nantes Cedex, France). Satisfactory quality of isotope measurement results was confirmed by obtained z-scores (−2.00 ≤ *z* ≤ 2.00). Both methods for isotopic measurements are accredited in accordance to HRN EN ISO/IEC 17025:2017 (72), which confirms laboratory ability to perform valid and comparable stable isotope results.

### Statistical Analysis

Results of isotopic and elemental analyses were uploaded to the software package Statistica 10.0 (Statsoft, Tulsa, Oklahoma, USA) and evaluated by descriptive statistical analysis (average values and standard deviations) and General Linear Model—Analysis of Variance (GLM-ANOVA) followed by *post-hoc* Tukey test and multivariate analysis methods. For statistical processing, elements with the values below the LOD were set to LOD/2 (73). Multivariate analysis was performed by principal component analysis (PCA) using the Unscrambler® software package, version 11.0 (CAMO AS, Norway) and general discriminant analysis (GDA) using the Statistica software package 10.0 (Statsoft, Tulsa, Oklahoma, USA). For visual presentation of results MS Excel® [Microsoft Office Professional Plus 2019, Microsoft Excel 2019 MSO (16.0.10354.20022)] was used.

## Results

All elements (Al, As, B, Ba, Ca, Cd, Co, Cr, Cu, Fe, K, Li, Mg, Mn, Mo, Na, Pb, Rb, Sn, Sr, V, and Zn) were analyzed by ICP-OES in appropriate linear calibration ranges (μg L^−1^ or mg L^−1^), which are presented together with limits of detection (LOD), limits of quantification (LOQ), recovery (%), and expanded measurement uncertainty (%) in Table 2. The achieved recovery was between 93% (for Al and Li) and 107% (for Mo and V). Assessment of expanded measurement uncertainty (with the coverage factor of *k* = 2, which gives a 95% confidence level for normal distribution) showed the highest expanded uncertainty for Mo (33%) and the lowest expanded uncertainty for Cd (4%).

GLM-ANOVA showed the significant interaction effect of the harvest year (*F* = 10.535; *p* < 0.001), type of production (*F* = 15.553; *p* < 0.001), and viticulture zone (*F* = 9.274; *p* < 0.001) on tested measurands (stable isotopes and elements). The effect of the type of production was significant for the harvest (*F* = 4.843; *p* < 0.001) and viticulture zones (*F* = 2.133; *p* < 0.001), and also the mutual interaction of these three effects was significant (*F* = 1.709; *p* < 0.005) indicating that these attributes were useful in characterizing the differences among the measured values in wines. The significance between the effects of harvest year and viticulture zones was not found.

The results of stable isotopes (δ^18^O and δ^13^C) and 22 elements measurements in a set of 190 Croatian wine samples are given in Table 3 according to the area of production (continental and coastal) and viticulture zones (B, CI, and CII) in Croatia, together with the GLM-ANOVA and *post-hoc* Tukey test results. The measurands with important significance found by GLM-ANOVA (*p* < 0.05) for the type of production (microvinified vs. commercial), harvest year (2015 vs. 2016), area (continental vs. coastal), viticulture zones B vs. CI, B vs. CII, and CI vs. CII are marked by numbers from 1 to 6, respectively. The average values of measured stable isotopes δ^18^O and δ^13^C were 1.37 ± 2.56‰ SMOW and −27.57 ± 1.47‰ V-PDB, respectively. The ICP-OES analyses results showed that Croatian wines contain elements that may contribute to the daily dietary intake of essential metals (i.e., copper, iron, and zinc) but can also have potentially toxic effects if metal concentrations are not kept under allowable limits. Analyzed wines contain also elements that have no nutritional value but are known to be potentially toxic, like arsenic, cadmium, and lead (74, 75). The results showed that the lowest concentration of all samples had the micro-elements Cd (0.7±1 μg L^−1^), Mo (4±2 μg L^−1^), Li (5±3 μg L^−1^), Co (6±4 μg L^−1^), and As (8±5 μg L^−1^). The highest concentrations had the macro-elements K (788±226 mg L^−1^), Ca (85±23 mg L^−1^), Mg (81±18 mg L^−1^), and Na (14±18 mg L^−1^). The determined concentrations of As, B, Cd, Cu, and Pb that are related to the safety of wines were within the acceptable limits established by the OIV—International Organization of Vine and Wine (76). Maximum permitted concentration (80 mg L^−1^) prescribed by the OIV was exceeded only for Na in three samples. The obtained results suggest that moderate consumption of Croatian wines may contribute to the daily dietary intake of essential minerals and trace elements without the danger of exceeding admissible daily dose or causing a toxic effect according to dietary reference values for nutrients of European Food Safety Authority (EFSA) (77).

**Table 3:** Summary of results (average values with standard deviations) for all measurands and samples according to the area of production (continental and coastal Croatia) and winegrowing zones (B, CI, and CII) in Croatia.

**Measurand** **(Unit)**	**GLM-ANOVA** **(*p* < 0.05)**^**a**^	**All samples** **(*n* = 190)**	**Continental Croatia** **= Zone B + Zone CI** **(*n* = 120)**	**Coastal Croatia** **= Zone CII** **(*n* = 70)**	**Zone B** **(*n* = 78)**	**Zone CI** **(*n* = 42)**
δ^18^O (‰ SMOW)	1, 2, 3, 4, 5, 6	1.37 ± 2.56	−0.22 ± 1.47	4.09 ± 1.52	−0.61 ± 1.44	0.51 ± 1.23
δ^13^C (‰ V-PDB)	2, 3, 5, 6	−27.57 ± 1.47	−28.31 ± 1.01	−26.29 ± 1.24	−28.38 ± 0.99	−28.20 ± 1.06
Al (mg L^−1^)	1, 3, 5	0.59 ± 0.52	0.51 ± 0.42	0.74 ± 0.64	0.48 ± 0.39	0.56 ± 0.48
As (μg L^−1^)		7.5 ± 5.4	7.2 ± 5.1	8.2 ± 5.9	7.2 ± 5.0	7.2 ± 5.2
B (mg L^−1^)	1, 3, 5, 6	2.98 ± 1.14	2.62 ± 1.03	3.60 ± 1.07	2.76 ± 0.97	2.36 ± 1.10
Ba (mg L^−1^)	6	0.11 ± 0.05	0.11 ± 0.05	0.10 ± 0.04	0.11 ± 0.05	0.12 ± 0.04
Ca (mg L^−1^)	1, 2, 3, 5	85.0 ± 22.7	89.3 ± 21.2	77.5 ± 23.6	92.1 ± 20.9	84.1 ± 20.9
Cd (μg L^−1^)	2	0.7 ± 1.0	0.7 ± 1.0	0.8 ± 0.8	0.7 ± 1.2	0.5 ± 0.7
Co (μg L^−1^)	2	5.9 ± 4.4	5.5 ± 3.9	6.6 ± 5.1	5.8 ± 4.4	4.9 ± 2.6
Cr (μg L^−1^)	2	19.0 ± 17.0	17.4 ± 12.0	21.8 ± 23.1	17.4 ± 11.8	17.4 ± 12.6
Cu (mg L^−1^)		0.18 ± 0.14	0.17 ± 0.15	0.18 ± 0.12	0.18 ± 0.17	0.16 ± 0.09
Fe (mg L^−1^)	1	1.91 ± 1.39	1.86 ± 1.43	20.1 ± 1.34	1.78 ± 1.32	2.00 ± 1.61
K (mg L^−1^)	3, 5, 6	788 ± 226	730 ± 180	889 ± 260	730 ± 179	730 ± 185
Li (μg L^−1^)	3, 6	4.6 ± 3.2	5.2 ± 3.5	3.6 ± 2.3	4.7 ± 3.3	6.0 ± 3.6
Mg (mg L^−1^)	1, 4, 5	81.3 ± 17.9	79.8 ± 16.6	83.9 ± 19.8	76.8 ± 15.9	85.4 ± 16.5
Mn (mg L^−1^)	4, 6	0.96 ± 0.52	1.01 ± 0.58	0.87 ± 0.39	0.92 ± 0.63	1.19 ± 0.45
Mo (μg L^−1^)	2	4.3 ± 2.1	4.4 ± 2.2	4.1 ± 2.0	4.7 ± 2.3	4.0 ± 2.0
Na (mg L^−1^)	1, 3, 5, 6	14.3 ± 18.1	10.6 ± 7.0	20.8 ± 27.3	10.7 ± 7.9	10.2 ± 5.0
Pb (μg L^−1^)	1, 2, 3	30.2 ± 18.7	28.0 ± 17.2	33.8 ± 20.5	29.1 ± 16.5	26.1 ± 18.6
Rb (mg L^−1^)	3, 5, 6	1.08 ± 0.41	0.99 ± 0.42	1.22 ± 0.35	1.05 ± 0.43	0.88 ± 0.37
Sn (μg L^−1^)	2, 3, 5, 6	55.0 ± 30.3	48.4 ± 31.1	66.3 ± 25.3	46.8 ± 29.2	51.6 ± 34.6
Sr (mg L^−1^)	1	0.46 ± 0.21	0.46 ± 0.17	0.46 ± 0.28	0.46 ± 0.18	0.45 ± 0.15
V (μg L^−1^)	1	83.6 ± 16.8	82.4 ± 15.8	85.6 ± 18.3	80.0 ± 15.1	86.7 ± 16.2
Zn (mg L^−1^)		0.69 ± 0.37	0.67 ± 0.32	0.73 ± 0.44	0.69 ± 0.34	0.64 ± 0.27

a *Measurands with p < 0.05 for: (1)—type of production; (2)—harvest year; (3)—continental and coastal area; (4)—zones B and CI; (5)—zones B and CII; (6)—zones CI and CII*.

A study of the data structure by PCA was carried out to aid in interpretation of the obtained data and to establish whether the wines from different wine producing geographical areas and viticulture zones constitute distinctive, well-defined groups. PCA was performed for all wines and variables (isotopes and elements) to determine whether different geographical regions of origin (areas and zones) had influenced the isotopes and elements profile. In this context, 2 isotopic ratios and 22 elements posed as the investigated variables, while wines posed as the cases under investigation.

PCA performed for entire dataset of microvinified and commercial wines (*n* = 190) is explaining only 65% of variability by first seven factors. The first two factors (PC1 and PC2) represent 29.2% of the initial data variability and 40% with the third factor (PC3). The remaining 25% of significant variability is hidden in the remaining four factors (PC4–PC7). Total variability of the first seven factors and eigenvectors of correlation matrix for all samples obtained by PCA is shown in Table 4.

**Table 4:** Eigenvectors (EV) of correlation matrix for all samples (*n* = 190) obtained by PCA for first seven factors (PC1–PC7 with eigenvalues of correlation matrix > 1), total variability % for calibration set (TV), total variability % obtained by cross validation (CV), and significance of the variables (highlighted are the *p* < 0.05).

	**PC1**		**PC2**		**PC3**		**PC4**		**PC5**		**PC6**		**PC7**	
TV (%)	15,64		13,52		10,87		7,43		6,41		5,82		5,15	
CV (%)	7,90		10,19		7,61		3,59		1,62		3,09		4,63	
**Measurand**	**EV**	***p***	**EV**	***p***	**EV**	***p***	**EV**	***p***	**EV**	***p***	**EV**	***p***	**EV**	***p***
^18^O/^16^O	0.08	0.03	0.04	0.06	−0.48	0.00	−0.15	0.00	0.20	0.00	0.09	0.23	−0.18	0.06
^13^C/^12^C	0.08	0.04	0.08	0.01	−0.46	0.00	0.04	0.44	0.27	0.00	0.06	0.46	−0.12	0.22
Al	0.28	0.00	−0.09	0.01	−0.09	0.02	−0.40	0.00	−0.14	0.11	0.07	0.44	0.26	0.08
As	0.08	0.09	−0.01	0.71	−0.08	0.02	−0.18	0.02	−0.26	0.00	−0.26	0.02	0.58	0.00
B	0.04	0.03	0.23	0.00	−0.29	0.00	0.10	0.00	−0.31	0.00	−0.31	0.00	−0.12	0.05
Ba	0.23	0.00	0.02	0.54	0.26	0.00	−0.15	0.06	0.28	0.00	−0.33	0.00	−0.17	0.04
Ca	0.11	0.01	0.24	0.00	0.27	0.00	0.24	0.00	−0.28	0.00	0.02	0.82	0.07	0.38
Cd	0.11	0.00	−0.24	0.00	−0.15	0.00	0.48	0.00	0.09	0.06	−0.04	0.42	0.18	0.01
Co	0.36	0.00	−0.29	0.00	−0.02	0.62	0.08	0.12	−0.05	0.21	−0.07	0.32	−0.12	0.26
Cr	0.22	0.00	−0.17	0.00	−0.10	0.29	0.02	0.82	−0.21	0.01	0.05	0.68	−0.35	0.06
Cu	0.01	0.92	0.00	0.98	0.03	0.65	0.07	0.64	−0.25	0.31	0.19	0.25	−0.36	0.40
Fe	0.13	0.05	−0.25	0.00	−0.07	0.30	−0.17	0.17	0.21	0.12	−0.03	0.86	0.18	0.49
K	0.11	0.01	0.16	0.00	−0.23	0.00	0.12	0.09	−0.07	0.21	−0.49	0.00	0.02	0.80
Li	0.22	0.00	−0.11	0.00	0.28	0.00	−0.20	0.03	0.08	0.42	−0.07	0.67	−0.15	0.23
Mg	0.28	0.00	0.34	0.00	0.03	0.13	0.19	0.00	0.24	0.00	0.09	0.04	0.11	0.16
Mn	0.28	0.00	0.01	0.49	0.25	0.00	−0.09	0.15	0.23	0.02	−0.23	0.08	−0.19	0.07
Mo	0.24	0.00	−0.21	0.00	0.06	0.15	0.33	0.00	−0.26	0.00	0.06	0.23	−0.07	0.45
Na	0.29	0.03	−0.02	0.59	−0.11	0.04	−0.27	0.12	−0.20	0.30	0.28	0.22	−0.02	0.88
Pb	0.24	0.00	−0.34	0.00	−0.18	0.00	0.17	0.00	0.08	0.11	0.03	0.64	0.14	0.17
Rb	0.03	0.53	0.18	0.00	−0.14	0.01	−0.19	0.04	−0.19	0.10	−0.36	0.01	−0.22	0.14
Sn	0.11	0.01	0.36	0.00	−0.10	0.02	−0.08	0.14	0.10	0.19	0.30	0.00	0.06	0.52
Sr	0.30	0.00	0.19	0.00	0.09	0.18	−0.01	0.92	−0.15	0.14	0.10	0.27	0.12	0.23
V	0.30	0.00	0.34	0.00	0.04	0.04	0.23	0.00	0.20	0.00	0.06	0.16	0.09	0.19
Zn	0.16	0.08	0.11	0.05	0.01	0.86	−0.11	0.47	−0.22	0.36	0.22	0.32	−0.03	0.92

The PCA model for entire data set was validated by segmented cross validation (random method, 20 segments, and 9 samples per segment) and significance of the variables (*p*-value) was estimated by a *t*-test. Most of the measurands were found to be significant (*p* < 0.05) for the differentiation of the geographical origin according to the obtained *p*-values (Table 4). Only Cu and Zn showed no significance in either of PC1–PC7. Because the calibration set (the raw data set, *n* = 190) explained only 65%, and the validation set correctly explained only 39% of total variability for first seven PCs (Table 4), it was concluded that overall uncertainty of the model is fairly high. Hence, the additional statistical tool of multivariate analysis (GDA) needed to be applied.

**Figure 2:**
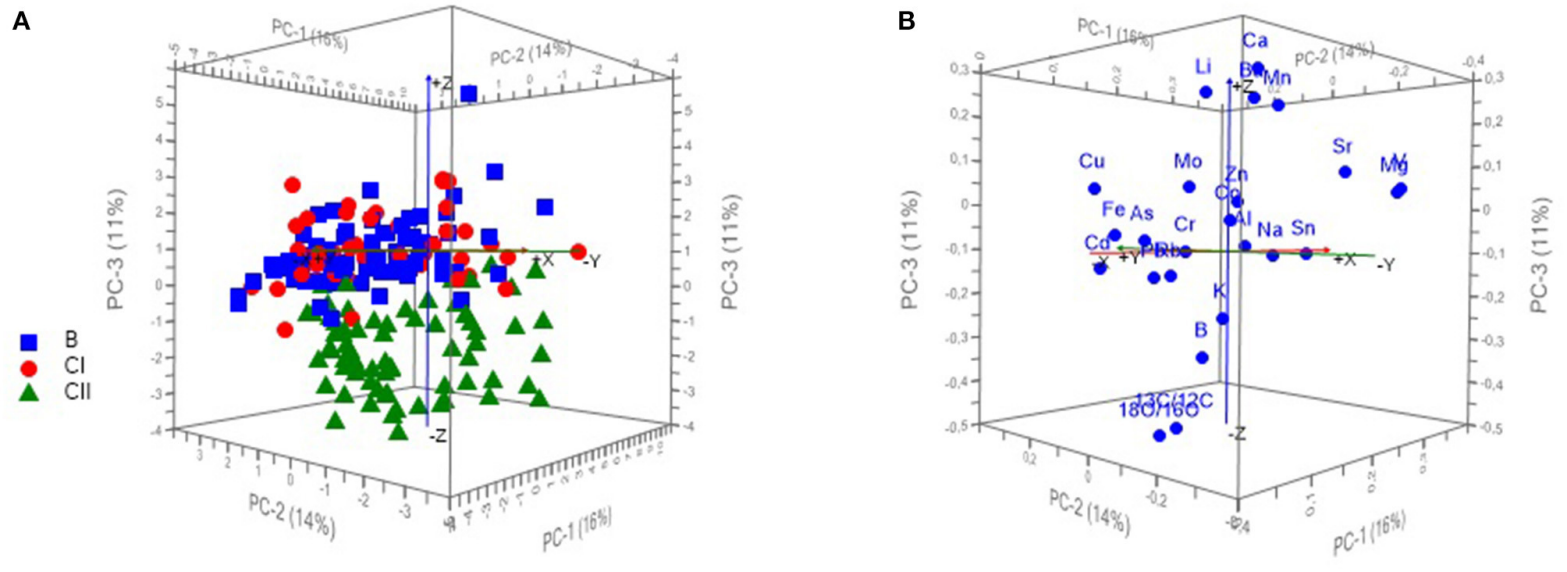
Scoreplot of PCA for 190 wine samples showing the projection of the cases (according to the viticulture zones) on the factor plane PC1 vs. PC2 vs. PC3 **(A)** and PCA loadings plot showing the position of 24 variables (measurands) on the plane PC1 vs. PC2 vs. PC3 **(B)**.

Scoreplot of PCA for 190 wine samples is showing the projection of the cases (according to the viticulture zones) on the factor planes PC1 vs. PC2 vs. PC3 (Figure 2A), where wines from the continental part of Croatia (zones B and CI) are positioned mostly on the positive side of PC3 while the wines from coastal Croatia (zone CII) remained on the negative side of the PC3. Positioning of the variables on the factor planes PC1 vs. PC2 vs. PC3 can be observed at Figure 2B indicating the strongest influence of δ^18^O and δ^13^C on the grouping of the samples from coastal Croatia.

Since microvinified samples are part of the Croatian national isotope database of authentic wines, PCA was also carried out to visualize the effect of the type of production on the positioning of the samples on the factor planes in order to establish whether the microvinified wines can be used as a representative set for the authenticity evaluation of declared geographical origin of commercial wines by used set of variables (stable isotopes and elements). In addition to the entire data set, the PCA was also performed for microvinified and commercial wines separately. Eigenvalues of correlation matrix (>1) showed that 76% of total variability is explained by the first eight factors for the microvinified, and 71% for commercial data set (Supplementary Table 1).

**Figure 3:**
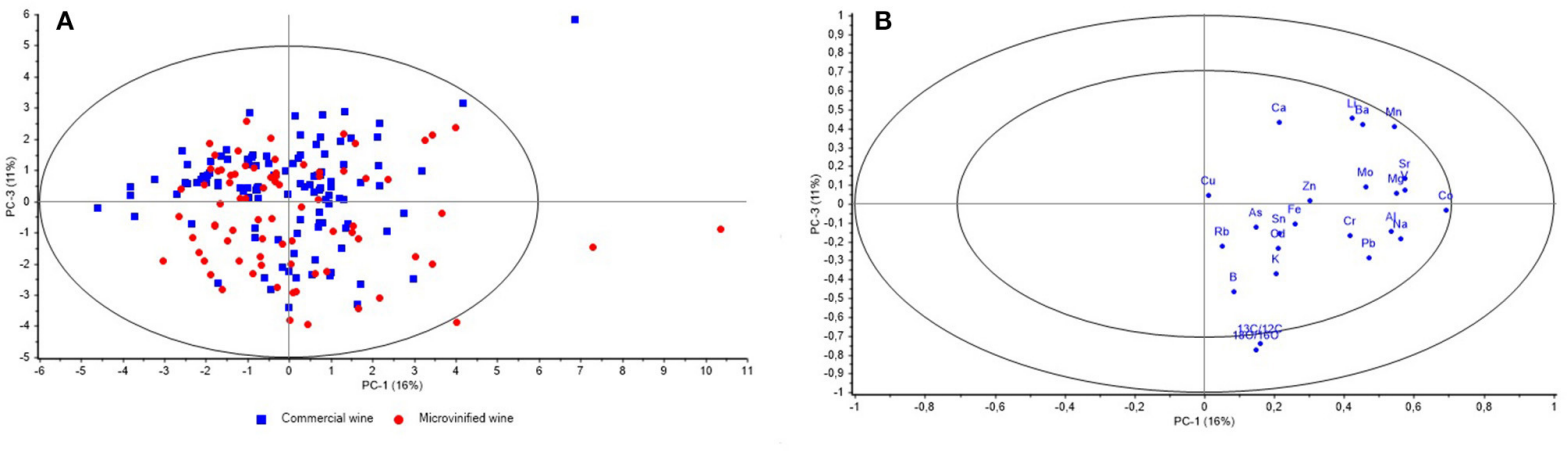
Scoreplot of PCA for 78 samples of microvinified wines and 112 samples of commercial wines showing the projection of the cases (according to the type of production) on the factor plane PC1 vs. PC3 **(A)** and PCA correlation loadings plot showing the position of 24 variables (measurands) on the plane PC1 vs. PC3 **(B)**.

The scoreplot of PCA for microvinified wines and commercial wines (Figure 3A) is showing the projection of the cases according to the type of production on the factor plane PC1 vs. PC3 and with the 95% confidence interval. Rather uniform distribution of microvinified and commercial samples in the PC1 and PC3 planes can be observed, indicating the same effect of the measured values (stable isotopes and elements) influencing the distribution of the samples, both microvinified and commercial. Figure 3B is showing PCA correlation loadings plot with the position of 24 variables (measurands) in the plane PC1 vs. PC3 and the Hotelling's T^2^ ellipse representing 50 and 100% of modeled variance (*r*^2^ = 0.5/1). The highest effect on the variability explained by PC3 have variables δ^18^O and δ^13^C positioned between the two ellipses.

GDA analysis was performed to choose the variable with the most significant contribution to the discrimination between continental and coastal winegrowing areas of Croatia and then for the viticulture zones B, CI, and CII. The reduced number of variables was used based on the significance obtained by PCA (Cu and Zn were omitted). Also, the model was validated through cross-validation using the set of microvinified wines as the model, and the set of commercial wines as unknown samples.

**Figure 4:**
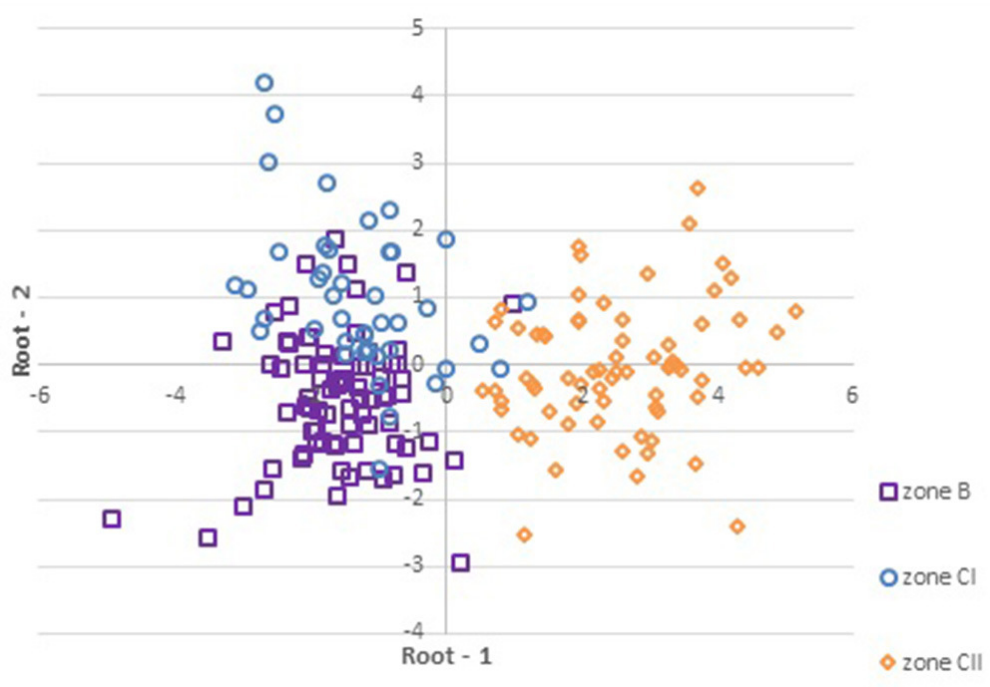
Projection of the scores (GDA) of the samples (*n* = 190) depending on zoning system in the plane defined by the two standardized canonical discriminant function coefficients (Root 1 and Root 2).

Figure 4 depicts the projection of the cases (zones) on the Root 1 vs. Root 2 where wines from the continental part of Croatia (zones B and CI) are positioned mostly on the negative side of root 1 while the wines from coastal Croatia (zone CII) remained on the positive side.

Multivariate test of significance (Wilks test, *p* ≤ 0.05; Supplementary Table 2) showed that most significant for geographical areas discrimination (coastal and continental) are δ^18^O and Co, followed by K, Rb, Sn, Li, and δ^13^C, and for discrimination of samples according to the zones most significant are δ^18^O and Co, followed by Rb, Li, K, and Sn, in descending order. Classification matrix obtained by GDA, showing the percentage of correctly predicted classifications (%) vs. the observed classifications for continental and coastal winegrowing areas and zones B, CI, and CII, is shown in Table 5. In the entire dataset (*n* = 190) the GDA classification matrix correctly classified 97.9% of the samples in regards to the winegrowing areas, while for the microvinified and commercial wines, correct classification was achieved for 100.0 and 84.8% of the samples, respectively.

**Table 5:** Classification matrix obtained by GDA showing the percentage of correctly predicted classifications (%) vs. the observed classifications for continental and coastal winegrowing areas and zones B, CI, and CII.

**Winegrowing areas** **and zones**	**Complete data** **set (all wines)** **(*n* = 190)**	**Authentic wines** **(*n* = 78)**	**Crossvalidation** **data set** **(commercial wines)** **(*n* = 112)**
Continental Croatia	97.5	100.0	87.2
Coastal Croatia	98.6	100.0	79.4
**Total**	**97.9**	**100.0**	**84.8**
Zone CII	100.0	100.0	79.4
Zone B	91.0	96.0	67.9
Zone CI	54.8	82.4	44.0
**Total**	**86.3**	**94.9**	**66.1**

Regarding the three viticulture zones, 86.3% of correct classification was achieved for the entire dataset, while for the microvinified and commercial wines, correct classification was achieved for 94.9 and 66.1% of the samples, respectively.

Regarding the differentiation between two zones (B and CI) of the continental part of Croatia, which can also be observed at the Figure 4 to some extent, GDA showed correct classification for 67.9% of the samples from zone B and for only 44.0% of the samples from zone CI. Correct classification was obtained for 79.4% of the CII zone samples.

## Discussion

The isotopic and multielement composition of the analyzed wines and statistical methods were used as chemical descriptors in order to establish criteria for wine classification and differentiation according to geographical origin.

The measurands with important significance found by GLM-ANOVA (*p* < 0.05) and marked from 1 to 6 in Table 3 are herein discussed in more detail. *Post-hoc* test (Tukey test) was conducted to evaluate the significance of the influence of the type of sample production, vintages, and winegrowing areas (continental and coastal Croatia), and viticulture climate zones (B, CI, and CII) on the measurands.

GLM-ANOVA of obtained results for all samples in regards to the type of production (Table 3, measurands denoted by 1) showed statistically significant differences between microvinified and commercial samples for δ^18^O, Al, B, Ca, Fe, Mg, Na, Pb, Sr, and V, which could be caused by oenological practices employed for production of commercial samples but lacking at the microvinification process, and by the differences in the size of actual samples (25 kg of grapes for microvinified vines). The δ^18^O values differences could also imply the mislabelling of the commercial samples in regard to the geographical origin or vintage, or possibility of water addition. Nevertheless, intensive rainfall during grape harvest also will be reflected in the isotope ratios values (13). To establish the possibility of fraudulent activities more elaborate investigation of isotopic ratios should be employed (23).

Regarding the harvest year (2015 and 2016), there were significant differences for δ^18^O, δ^13^C, Ca, Cd, Co, Cr, Mo, Pb, and Sn (Table 3, measurands denoted by 2), showing the contribution of the seasonal meteorological conditions influencing their uptake (5, 78). δ^18^O of water values were more positive (in average for 1‰) in the 2015 than in the 2016 harvest. This can be explained by the higher rainfall in September during the 2016 harvest (average 152 mm) (79) compared to the 2015 harvest (average 88 mm) (80). Similar influence of rainfall on δ^18^O of wine water was observed by previous research (13, 56). Variations of δ^13^C can also be the result of plant growth conditions, which can significantly modify 13C isotope values (81), in particular, the use of CO_2_ from photorespiration by the plant that reacts to water deficit by closing the stomata (82).

Regardless of the studied vintage year or the type of production, the GLM-ANOVA of isotopic ratios and multielement content enabled the discrimination of the two studied winegrowing areas (continental and coastal Croatia) and three winegrowing zones (B, CI, and CII). The statistically significant discrimination of the continental and coastal winegrowing areas was achieved for the following measurands: δ^18^O, δ^13^C, Al, B, Ca, K, Li, Na, Pb, Rb, and Sn (Table 3, measurands denoted by 3). These measurands were also identified as the key explanatory factors in various combinations for geographical origin determination by other researches, i.e., for Spanish (40, 41), Italian (34, 35), Romanian (53, 83, 84), Cypriot (7), USA (85), Brazilian (56), or Chinese (57) wines.

Wine samples from continental vineyards presented significantly lower average values of δ^18^O than those from coastal vineyards (−0.2 and 4.1‰ SMOW, respectively). These differences between geographical areas can be explained by the specific climatic conditions of each individual area, such as temperature, humidity, as well as meteorological conditions. The mean values of δ^18^O found in this research are consistent to those obtained for Croatian wines of vintages 1999–2001 (24). Obtained δ^18^O values are also in accordance with the wines from different European regions (22). The range of δ^13^C values of wines from the two investigated geographical areas is variating from −26.3‰ V-PDB in coastal part of Croatia to −28.3‰ V-PDB in continental area. The mean values of δ^13^C found in the present work are similar to those obtained by previous research (22, 24).

Samples from the coastal Croatian vineyards had significantly higher content of Al, B, K, Na, Pb, Rb, and Sn than the continental vineyards. The values of Na were almost double in coastal (21 mg L^−1^) than in continental areas (11 mg L^−1^) due to the proximity of the Adriatic Sea. This influence of the sea on the elevated Na content was observed by other investigations (7, 86, 87). As opposed to this, the continental vineyards were characterized by higher levels of Ca (89 mg L^−1^) and Li (5 μg L^−1^) than in coastal vineyards (76 mg L^−1^ of Ca and 4 μg L^−1^ of Li).

Statistically significant discrimination between two continental winegrowing zones B and CI is achieved only by the δ^18^O, Mg, and Mn (Table 3, measurands denoted by 4). As expected with regards to geographical and climatic conditions, average values of δ^18^O of wine water from the eastern continental part of Croatia (zone CI) were higher than those of the wines from vineyards in the western continental region of Croatia (zone B), 0.51 and −0.61‰ SMOW, respectively. Both elements, the Mg and Mn, had higher content in zone CI (85 and 77 mg L^−1^, respectively) than in zone B (1.2 and 0.9 mg L^−1^, respectively).

In regards to the differentiation of the coastal zone CII vs. continental zone B, significant were the same measurands (Table 3, measurands denoted by 5) as for entire costal vs. continental area, with the exception of Pb and the addition of Mg, which was able to discriminate between zones CII and B (84 and 77 mg L^−1^, respectively).

δ^18^O, δ^13^C, B, Ba, K, Li, Mn, Na, Rb, and Sn (Table 3, measurands denoted by 6) enabled the differentiation of the coastal zone CII vs. continental zone CI. Average values of element B were significantly higher in coastal zone CII (3.6 mg L^−1^) than in continental zone CI (2.4 mg L^−1^ respectively). Ba was found to be significant only in discrimination of zones CI and CII, but it also enabled the geographical origin differentiation in the research of Croatian (37), Italian (35), Romanian (42), and South African (31) wines.

Compared to the PCA results for the entire data set, which explains 65% of the variability, the set of microvinified samples has a higher percentage of explained variability (75%) and better presents the geographic origin than the whole data set (Supplementary Table 1). This difference can be explained by the fact that microvinified samples do not have the influence of elements from the production process, i.e., Al, B, Cu, K, Fe, Mn (88–91). In these samples, the distinction of geographical origin is achieved only by endogenous measurands that reached the wine naturally, i.e., stable isotopes (5) or elements Mg, Sr (57, 88) and/or as natural contaminant such as Na (91). Even commercial samples evaluated separately by PCA have explained more variability (71%) than the whole set (Supplementary Table 1). This can also be explained by the influence of a technological process that is more or less similar in all commercial samples. Hence, it can be concluded that the combination of samples of different types of production leads to less explained overall variability.

As seen at Figure 2A, the 3D representation of the samples obtained from PCA using the raw data matrix (190 samples and 24 measurands) and the first three components indicates a satisfactory separation of samples according to the geographical area, although the first three components explained only 40% of the total variation. The samples from continental Croatia (zone CII) are well-distinguished from the samples from coastal Croatia (zones B and CI). The differentiation of continental zones B and CI by PCA method was not achieved.

It is shown that δ^18^O and δ^13^C (Figure 2B) have the strongest influence on separation of the CII zone from B and CI zones in the plane PC1 vs. PC2 vs. PC3 (Figure 2A). The significance of this influence is also visible at the Figure 3B, which is showing Hotelling's T^2^ ellipse representing 50 and 100% of modeled variance (*r*^2^ = 0.5/1). The highest effect on the variability explained by PC3 have variables δ^18^O and δ^13^C positioned between the two ellipses.

Overlapping of microvinified and commercial samples in the PC1 vs. PC3 planes (Figure 3A) is showing similar impact of the measured values on observed variability. This is an indication that the microvinified wines can be used as a representative set for the authenticity evaluation of declared geographical origin of commercial wines by used set of variables (stable isotopes and elements). However, the positioning of three microvinified samples from CII zone and one commercial sample from zone B outside of the 95% confidence interval can be noticed. This could be the result of specific microclimatic and pedologic characteristics of individual vineyards locations and it can be supported by the research of Croatian winegrowing regions (92) where it was found that both Western and Eastern continental Croatian regions, ranging from 1,323.9 to 1,652.5 GDD for the observed climatologic period (1988–2017) belong to the Winkler Regions I and II (zone A and B). In regards to Coastal Croatia in the same period, values ranged from 1,496.5 to 2,483.5 GDD, which is Winkler Region II to V (zones B, CI, CII, and CIII). The reason for outlying of the commercial sample from the zone B should be explored in more detail, considering all relevant meteorological and winegrowing parameters such as precipitation, harvest date, grape variety, and to use a representative number of reference samples (23).

GDA was found to be the most distinguishing chemometric tool for discrimination of Croatian wines according to the area of geographical origin. As seen at Table 5, the highest power for discrimination of wines produced in coastal and continental Croatia showed GDA by correct classification 100.0% of microvinified samples, 97.9% of all investigated samples, and 84.8% of commercial samples in the cross-validation matrix.

GDA showed somewhat weaker separation (Figure 4) of the zones B and CI in comparison to the excellent discrimination of continental and coastal areas. This can be explained by incompliance between official borders of the zones (Figure 1) (63) and actual situation presented by previous research (92), which established that within the zone B exist a smaller area corresponding to the Winkler Region I (zone A) and that Slavonia and Croatian Danube fall into Region II, which is zone B and not CI as stated by the current EU division system.

This can explain the deviation of some samples outside of the designated zones in particular if taking into consideration that the most dominant marker of the geographical origin identified by this research is the δ^18^O, which is also strongly influenced by the climate (5, 22).

The analysis of bioclimatic indices in Croatian winegrowing regions (92) would enable more accurate interpretation of isotopic and multielement data found in this research as the tools for Croatian wine geographical origin determination. Furthermore, current administrative division of the zones established by the EU legislation (63) is defining the limits and conditions for certain oenological practices (enrichment limits/increase in the natural alcoholic strength) where climatic conditions have made it necessary in certain winegrowing zones. Consequently, a question arises of interpreting the isotopic data from EU wine data bank in regards to chaptalization, requiring a larger number of representative samples and expert interpretation. The shortcomings of Croatian vineyards zoning are also suggested by projections of further warming and drying of the climate in Croatia (93), making the existing viticulture zoning even less adequate.

This study verified that stable isotopes of oxygen and carbon have proven to be most valuable indicators of discrimination of wines from Croatian winegrowing areas and zones and especially in the combination with the multielemental composition analysis, which was conducted here for the first time for Croatian wines.

Results suggest that the proposed methodology is a powerful tool and it could add extra value to local Croatian wines by emphasizing the wine authenticity importance, especially in the light of the growing tourism industry and increasing awareness of winemaking significance as economic activity.

## Data Availability Statement

The raw data supporting the conclusions of this manuscript will be made available by the authors, without undue reservation, to any qualified researcher.

## Author Contributions

RL and MB: conceptualization. RL and IVP: methodology and writing-original draft preparation. JJ and RL: formal analysis and investigation. MB: writing-review and editing and supervision. All authors: contributed to the article and approved the submitted version.

## Conflict of Interest

JJ was employed by Department of Physico-Chemical Testing, Center for Viticulture, Enology and Edible Oils Analysis, Croatian Agency Agriculture and Food, Zagreb, Croatia, during the research and as she performed the ICP-OES analyses. After the experimental part of the research was finished, she changed her job and her current employment is at company Primevigilance d.o.o., Zagreb, Croatia. The remaining authors declare that the research was conducted in the absence of any commercial or financial relationships that could be construed as a potential conflict of interest.
