# Supplementary material for: Geographical Discrimination of Croatian Wines by Stable Isotope Ratios and Multielemental Composition Analysis
Source: Front Nutr. 2021 Mar 4;8:625613. doi: 10.3389/fnut.2021.625613 (PMC7982904; doi:10.3389/fnut.2021.625613)
Supplement: Supplementary file 1 [file Data_Sheet_1.PDF]

Table S1. PC1 – PC8 with eigenvalues of correlation matrix > 1, total variance, cumulative eigenvalue and cumulative variance for microvinified, commecial and all samples.

**Eigenvalues of correlation matrix, and related statistics**				
	Eigenvalue	Total variance %	Cumulative Eigenvalue	Cumulative %
**Microvinified samples, n = 78**				
PC1	5,45	22,69	5,45	22,69
PC2	3,37	14,02	8,81	36,72
PC3	2,54	10,58	11,35	47,30
PC4	1,82	7,57	13,17	54,87
PC5	1,52	6,34	14,69	61,20
PC6	1,41	5,86	16,10	67,07
PC7	1,14	4,76	17,24	71,82
PC8	1,02	4,25	18,26	76,07
**Commercial samples, n = 112**				
PC1	4,19	17,46	4,19	17,46
PC2	3,31	13,78	7,50	31,25
PC3	2,50	10,41	10,00	41,66
PC4	1,86	7,74	11,86	49,40
PC5	1,56	6,50	13,42	55,90
PC6	1,30	5,40	14,71	61,30
PC7	1,21	5,03	15,92	66,33
PC8	1,07	4,47	16,99	70,80
**All samples, n = 190**				
PC1	3,75	15,64	3,75	15,64
PC2	3,24	13,52	7,00	29,16
PC3	2,61	10,87	9,61	40,03
PC4	1,78	7,43	11,39	47,46
PC5	1,54	6,41	12,93	53,87
PC6	1,40	5,82	14,33	59,69
PC7	1,24	5,15	15,56	64,85
