# Supplementary material for: Geographical Discrimination of Croatian Wines by Stable Isotope Ratios and Multielemental Composition Analysis
Source: Front Nutr. 2021 Mar 4;8:625613. doi: 10.3389/fnut.2021.625613 (PMC7982904; doi:10.3389/fnut.2021.625613)
Supplement: Supplementary file 2 [file Data_Sheet_2.PDF]

Table S2. Multivariate test of significance (Wilks test; highlighted are the p-values < 0,05) of effects (measures values) on the geographical area (Continental and Coastal) and viticulture zone (C, CI and CII).

Multivariate Tests of Significance - Wilks test										
Effect	Coastal and Continental area					zone B, CI and CII				
	Value	F	Effect df	Error df	p	Value	F	Effect df	Error df	p
Intercept	0,988	1,997	1	167	0,160	0,972	2,361	2	166	0,097
18O/16O	0,591	115,400	1	167	0,000	0,571	62,404	2	166	0,000
13C/12C	0,977	4,003	1	167	0,047	0,973	2,270	2	166	0,107
Al	0,992	1,376	1	167	0,243	0,989	0,952	2	166	0,388
As	1,000	0,057	1	167	0,812	0,999	0,118	2	166	0,889
B	0,994	1,053	1	167	0,306	0,992	0,642	2	166	0,528
Ba	0,999	0,093	1	167	0,761	0,997	0,232	2	166	0,793
Ca	1,000	0,037	1	167	0,849	0,981	1,603	2	166	0,204
Cd	1,000	0,006	1	167	0,940	0,991	0,777	2	166	0,461
Co	0,925	13,583	1	167	0,000	0,895	9,725	2	166	0,000
Cr	0,993	1,122	1	167	0,291	0,985	1,280	2	166	0,281
Fe	1,000	0,045	1	167	0,832	0,998	0,190	2	166	0,827
K	0,946	9,539	1	167	0,002	0,944	4,968	2	166	0,008
Li	0,959	7,110	1	167	0,008	0,928	6,483	2	166	0,002
Mg	0,999	0,223	1	167	0,638	0,994	0,465	2	166	0,629
Mn	0,982	3,054	1	167	0,082	0,973	2,299	2	166	0,104
Mo	1,000	0,077	1	167	0,782	0,999	0,103	2	166	0,902
Na	1,000	0,029	1	167	0,866	1,000	0,016	2	166	0,984
Pb	0,997	0,424	1	167	0,516	0,988	0,986	2	166	0,375
Rb	0,947	9,408	1	167	0,003	0,917	7,502	2	166	0,001
Sn	0,952	8,420	1	167	0,004	0,949	4,431	2	166	0,013
Sr	0,999	0,155	1	167	0,694	0,996	0,353	2	166	0,703
V	1,000	0,053	1	167	0,818	1,000	0,040	2	166	0,961
